# A randomized controlled clinical trial of prolonged balloon inflation during stent deployment strategy in primary percutaneous coronary intervention for ST-segment elevation myocardial infarction: a pilot study

**DOI:** 10.1186/s12872-022-02477-0

**Published:** 2022-02-04

**Authors:** Min Ma, Ling Wang, Kai-yue Diao, Shi-chu Liang, Ye Zhu, Hua Wang, Mian Wang, Li Zhang, Zhi-gang Yang, Yong He

**Affiliations:** 1 Department of Cardiology, West China Hospital, Sichuan University, No. 37 GuoXueXiang, Chengdu, 610041 China; 2 Department of Cardiology, The Sixth People’s Hospital of Chengdu, Chengdu, China; 3 Department of Cardiology, Mian Yang People’s Hospital, Chengdu, China; 4 Department of Radiology, State Key Laboratory of Biotherapy, West China Hospital, Sichuan University, No. 37 Guoxue Street, Chengdu, 610041 China

**Keywords:** Prolonged inflation strategy, Rapid inflation/deflation strategy, ST-segment elevation myocardial infarction, Randomized controlled trial

## Abstract

**Background:**

Primary percutaneous coronary intervention (PPCI) is the standard procedure for reperfusion for ST-segment elevation myocardial infarction (STEMI), but the occurrence of the no-reflow phenomenon remains common and is associated with adverse outcomes.

**Aims:**

This study aimed to evaluate whether prolonged balloon inflation in stent deployment would lessen the occurrence of the no-reflow phenomenon in PPCI compared with conventional rapid inflation/deflation strategy.

**Methods:**

Patients were randomly assigned to either the prolonged balloon inflation in stent deployment group (PBSG) or conventional deployment strategy group (CDSG) in a 1:1 ratio. A subset of patients was included in the cardiac magnetic resonance (CMR) assessment.

**Results:**

Thrombolysis in MI (TIMI) flow grade 3 was found in 96.7% and 63.3% of the patients of the PBSG and CDSG, respectively (*P* = 0.005). The results of the PBSG and CDSG are respectively shown as follows: 0% versus 30% no-reflow or slow flow (*P* = 0.002); 90% versus 66.7% ST-segment resolution ≥ 50% (*P* = 0.028); 35.6 ± 14.5 frames versus 49.18 ± 25.2 frames on corrected TIMI frame count (*P* = 0.014); and 60% versus 20% myocardial blush grade 3 (*P* = 0.001). At 1 month, the major cardiovascular adverse event (cardiovascular mortality) rate was 3.3% in both groups; at 1 year, the rate was 3.3% and 6.7% for the PBSG and CDSG, respectively (*P* = 1.00). In the CMR subset of cases, the presence of microvascular obstruction (MVO) was detected in 6.7% and 50% of the patients in the PBSG and CDSG, respectively (*P* = 0.023).

**Conclusion:**

In our pilot trial, prolonged balloon inflation during stent deployment strategy in PPCI reduces the occurrence of the no-reflow phenomenon in patients with STEMI and improved the myocardial microcirculation perfusion (ClinicalTrials.gov number: NCT03199014; registered: 26/June/2017).

## Background

Although the use of primary percutaneous coronary intervention (PPCI) for ST-segment elevation myocardial infarction (STEMI) in China has increased in recent decades, the in-hospital mortality and long-term prognosis of patients has not significantly changed [1]. Coronary no-reflow is defined as a phenomenon in which, despite revascularization therapy with PPCI, the cardiac tissue fails to normally perfuse and has a negative effect on outcomes, negating the potential benefits of PPCI [2–5].

The pathophysiology of the no-reflow phenomenon is multifactorial, and it is now referred to as microvascular obstruction (MVO), which includes injuries related to ischemia, reperfusion, endothelial cell edema, thrombus embolization, and embolization of atherosclerotic plaque fragments [6]. Therefore, these injuries are of concern during PPCI [7]. In theory, the manipulation of factors during PPCI may contribute to no-reflow; however, the balloon pressure, inflation time, repeated balloon dilations, and stent number did not affect the incidence of no-reflow in a retrospective analysis [8]. In small vessels, it is possible that the stent itself affixes the thrombus to the vessel wall, especially if the thrombus is more fibrous [9].

The duration of stent inflation has a significant impact on stent expansion, and rapid inflation/deflation may not be sufficient to fully expand the stent [10, 11]. Therefore, a fully expanded stent may entrap the atherothrombus under the struts that are more fixed and reduce distal embolization in PPCI. However, this hypothesis has not been thoroughly investigated in randomized controlled trial (RCT) studies. The present study was undertaken to evaluate whether prolonged balloon inflation during stent deployment strategy would lessen the occurrence of the no-reflow phenomenon in PPCI compared with the conventional rapid inflation/deflation strategy.

## Materials and methods

### Trial design and study participants

We conducted this single-center, randomized, single-blinded, parallel pilot trial to compare prolonged balloon inflation during stent deployment group (PBSG) (the stent was deployed with a single balloon inflation, and low-pressure inflation was sustained for > 30 s after the target balloon inflation pressure had been achieved. If the patient could not tolerate the procedure, showed signs of ischemia, or had chest pain, arrhythmia, or a decrease in blood pressure, the stent balloon was immediately deflated) with the conventional deployment strategy group (CDSG) (as a control group, in which the stent balloon inflation time was less than 10 s. Additional dilation, expansion pressure, and post dilation were left to the discretion of operators). Randomization was performed using a simple random sampling method generated by a computer program. The random allocation list was enclosed in sequentially numbered, opaque, and sealed envelopes. The eligible patients were assigned to the PBSG or CDSG in a 1:1 ratio. The trial design was approved by the Regional Ethics Review Board of West China Hospital. The research paper is written in accordance with the international CONSORT 2010 statement [12].

The patients were eligible for enrollment if they met the following criteria: (1) age ≥ 18 years; (2) patients with STEMI who were referred to PPCI within 12 h after the onset of symptoms and with ST-segment elevation ≥ 1 mm in ≥ 2 contiguous leads or a presumed new left bundle branch block or a true posterior MI; and (3) admission within 12 h of symptom onset or admission between 12 and 24 h if there was evidence of continuing ischemia. The exclusion criteria were as follows: cardiogenic shock, an intra-aortic balloon pump implant, or extracorporeal membrane oxygenation; thrombolysis; previous myocardial infarction; severe valvular disease; coronary artery bypass grafting; renal dysfunction (glomerular filtration rate of < 30 mL/min/1.73 m^2^); cardiomyopathy; congenital heart disease; malignant arrhythmia; chronic obstructive pulmonary disease; pregnancy; a contraindication to cardiac magnetic resonance (CMR) imaging (e.g., pacemaker and claustrophobia); and inability to give informed consent.

### Procedures

Following coronary angiography (CAG), the patients who fulfilled the inclusion criteria were randomly assigned to PBSG or CDSG during PPCI. All patients received 300 mg oral aspirin and 600 mg clopidogrel (or 180 mg ticagrelor) as soon as STEMI was confirmed. The prescription of anticoagulants, glycoprotein IIb/IIIa inhibitors, and thrombus aspiration was left to the physician’s discretion. The patients were immediately taken to the cardiac catheterization lab. If the operator predicted that direct stenting was possible, predilation was not performed. If direct stenting was impossible, predilation with a single low-pressure inflation was performed. In all patients, only the infarct-related artery was treated. All procedures undergone by the patients in the study were performed by experienced cardiologists who were trained by the National Coronary Intervention Training Bases. All operations were performed according to the standardized procedures. Less than 10% residual stenosis was considered as the ideal stent implantation.

All placed stents were drug-eluting stents, and the patients were given the option to choose between two commercially available drug-eluting stents: the PROMUS Element stent (Boston Scientific, Boston, Massachusetts, USA) or a GuReater stent (Lepu, China). The diameters of the stents and balloons were selected by visual estimation to achieve a balloon/vessel ratio of 1:1 for both study groups. After stent implantation, if the no-reflow phenomenon occurred, pharmacological intervention, including common vasodilators (nitroprusside and/or diltiazem), was administered.

### End points, assessment of outcomes, and definitions

The primary outcome of this study was the incidence of the no-reflow phenomenon according to thrombolysis in MI (TIMI) flow grade, a grading system used to evaluate blood flow. TIMI 0 is defined as no antegrade flow beyond the point of occlusion. TIMI 1 is defined as faint antegrade flow beyond the occlusion with incomplete filling of the distal vascular bed. TIMI 2 is defined as delayed antegrade flow with complete filling of the distal vascular bed. TIMI 3 is defined as normal flow with complete filling of the distal vascular bed. The no-reflow phenomenon was defined as TIMI flow grade < 3 and as myocardial blushing grade (MBG) < 2. The MBG is used to assess the filling and clearance of contracts in the myocardium. MBG 0 is defined as no apparent tissue-level perfusion in the distribution of the culprit artery. MBG 1 is defined as no clearance from the microvasculature. MBG 2 is defined as blush beginning to clear during washout [13], and MBG 3 is defined as normal myocardial blush or contrast density, comparable with that obtained during angiography of a contralateral or ipsilateral non-infarct-related coronary artery. The patients with no-reflow had serious outcomes, characterized by a significant increase in the incidence of congestive heart failure, cardiogenic shock, and death [14]. Two experienced cardiologists independently assessed the occurrence of no-reflow after stent implantation, TIMI flow grade, TIMI frame count, and MBG on the most recent angiogram. The number of angiogram frames required for the dye to reach a specified distal segment in the coronary artery was referred to as the corrected TIMI frame count [15]. All angiograms were recorded on a 21-mm cine film at 15 frames/second (UNIQ FD 10, Philips, USA). The thrombus burden was evaluated by the TIMI thrombus scale, which was classified as a grade between 0 and 5 [16]. The electrocardiographic resolution of the ST-segment elevation was defined as a reduction of > 50% of the ST-segment elevation in the same lead within 60 min after the index procedure [17].

The secondary outcomes were major adverse cardiovascular events, which were defined as any events of target vessel revascularization (TVR), recurrent MI, or cardiovascular mortality. These outcomes were examined 30 days and 1 year after the PPCI. The procedure time, total fluoroscopy time, and radiation dose were also assessed. The safety outcome of major bleeding was defined using a definition of major bleeding from the International Society on Thrombosis and Haemostasis [18]; the bleeding events that were not considered as major were considered as minor bleeding.

A subset of patients was included in the CMR examination approximately 3–5 days after the index procedure. The criteria among those who received CMR were infarct-related artery ≥ 2.5 mm in diameter and age > 18 years who provided informed consent was obtained for CMR. The exclusion criteria were as follows: cardiogenic shock, an intra-aortic balloon pump implant, or extracorporeal membrane oxygenation; thrombolysis; previous myocardial infarction; severe valvular disease; coronary artery bypass grafting; renal dysfunction (glomerular filtration rate of < 30 mL/min/1.73 m^2^); cardiomyopathy; congenital heart disease; malignant arrhythmia; chronic obstructive pulmonary disease; pregnancy; a contraindication to cardiac magnetic resonance (CMR) imaging (e.g., pacemaker and claustrophobia); and inability to give informed consent. Next, the infarct size, myocardial salvage index, presence and extent of MVO, myocardium hemorrhage, myocardial edema, and left ventricular ejection fraction (LVEF, was calculated by subtracting the volume at end systole from the volume at end diastole and dividing the result by the volume at end diastole) and left ventricular (LV) volume were assessed. The examinations were performed on a 3.0-T whole-body scanner with an 18-element body phased array coil (Skyra; Siemens Medical Solutions, Erlangen, Germany). Standard two-, three-, and four-chamber cine images were acquired using a TrueFISP sequence. The area at risk was assessed on the initial examination using a T2-weighted short-tau inversion recovery sequence. The infarct size, LVEF, and LV volume were assessed on both examinations using delayed, contrast-enhanced, electrocardiogram-triggered inversion recovery images and steady-state free precession cine images. The myocardial salvage index was calculated as [area at risk (mass) − infarct size (mass)]/area at risk (mass). The delayed contrast-enhanced images were obtained 10 min after an intravenous injection of 0.1 mmol/kg body weight gadolinium-based contrasts (Gadovist, Bayer Schering, Berlin, Germany). All images were obtained in the short-axis plane, with 8-mm slices without gaps covering the entire LV [19–21].

A subgroup analysis was prespecified to examine the factors associated with an increased risk of the no-reflow phenomenon, which were comprised of the following: age (≥ 65 and < 65 years), thrombus burden, thrombosis aspiration, direct stenting, infarct location (anterior and nonanterior), multiple complex lesions, and door-to-balloon time.

### Statistical analysis

On the basis of the previous research, no-reflow phenomenon was observed in 32% of the patients following PPCI for acute MI (AMI). No-reflow was defined as TIMI < 3 and as MBG < 2. Some studies on the current routine interventions for the no-reflow phenomenon in STEMI, such as the REOPEN-AMI study, showed that the additional intracoronary administration of adenosine results in a significantly reduced no-reflow by 12%, which was similar to the TAPAS study in which thrombus aspiration was evaluated. We calculated that a sample size of 53 patients in each group would provide the trial with a power of at least 80% and a two-side alpha of 5% to reject the null hypothesis of no difference between the groups, assuming that the adopted relative risk (RR) reduction of the primary outcome was 22% in the PBSG. Under an assumption that about 20% of the patients would be lost to follow, a total of 120 patients was deemed to be sufficient to evaluate the primary outcome. The data are expressed as percentages and means (standard deviations). The categorical variables were compared using the Chi-square test at a two-sided significance level of 5%. The continuous variables were compared using t test or analysis of variance. A *P* value < 0.05 was considered statistically significant. All statistical analyses were performed using the SPSS 17.0 software (SPSS Inc., Chicago, Illinois, USA).

## Result

### Patient characteristics

**Fig. 1:**
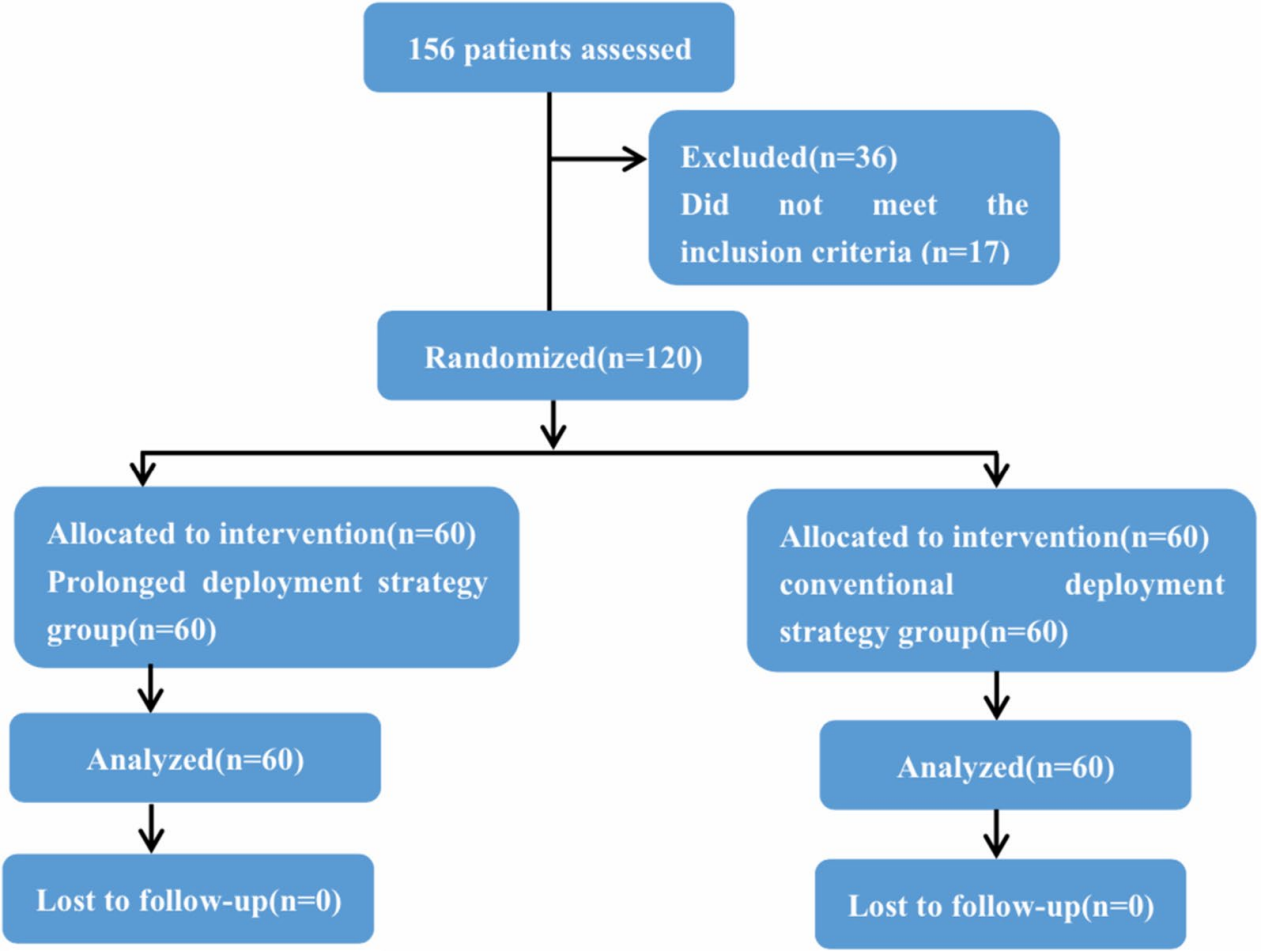
Study flow chart

Between November 2016 and May 2018, 156 patients with STEMI were considered for inclusion, of which 120 were enrolled according to the eligibility criteria (Fig. 1). After CAG, the patients were randomly assigned into either PBSG (n = 60) or CDSG (n = 60) during PPCI. The baseline clinical characteristics were similar in both groups (Table 1). The procedural and angiographic characteristics of the study patients are shown in Table 1. After undergoing randomization, the mean inflation time was 35.87 and 10.07 s in the PBSG and CDSG, respectively. None of the patients who underwent randomization were lost to follow-up.

**Table 1:** Baseline characteristics of the patients according to the randomization allocation

Characteristic	PBSG (n = 60)	CDSG (n = 60)	*P* value
Age-years	64.2 ± 10.9	61.9 ± 12.5	0.445
Male sex-no. (%)	50	44	0.347
Body mass index	25.0 ± 2.8	24.5 ± 3.7	0.602
History			
Hypertension-no. (%)	28 (46.7)	34 (56.7)	0.438
Current smoking-no. (%)	44 (73.3)	34 (56.7)	0.176
Previous myocardial infarction	0 (0)	0 (0)	NA
Diabetes mellitus-no. (%)	12 (20)	8 (13.3)	0.488
Previous stroke-no. (%)	8 (13.3)	4 (6.7)	0.389
Previous atrial fibrillation-no. (%)	2 (3.3)	2 (3.3)	1.000
Medication used			
ACEI-no. (%)	0 (0)	1 (3.3)	0.313
ARB-no. (%)	4 (6.7)	2 (3.3)	0.554
CCB-no. (%)	8 (13.3)	10 (16.7)	0.718
Metformin-no. (%)	2 (3.3)	4 (6.7)	0.554
β-blocker-no. (%)	0	4 (6.7)	0.492
Clopidogrel-no. (%)	44 (73.3)	40 (66.7)	0.779
Ticagrelor-no. (%)	16 (26.7)	20 (33.3)	0.779
Fibrinolysis before randomization-no. (%)	0 (0)	4 (6.7)	0.492
Door-to-balloon time-min	67 ± 17	67 ± 25	0.972
Pain onset to reperfusion-hour	7.6 ± 2.3	7.5 ± 2.1	0.651
Target coronary artery			0.663
Left coronary artery-no. (%)	28 (46.7)	24 (40)	
Left circumflex-no. (%)	4 (6.7)	8 (13.3)	
Right coronary artery-no. (%)	28 (46.7)	28 (46.7)	
Multivessel coronary disease-no. (%)	30 (50)	30 (50)	1.000
Killip class-no. (%)			0.431
1	54	52	
2	4	8	
3	2	0	
4	0	0	
Systolic blood pressure in lab-mmHg	125.4 ± 21.0	120.2 ± 21.2	0.359
Diastolic blood pressure in lab-mmHg	79.8 ± 16.6	75.7 ± 13.7	0.301
Heart rate in lab-bpm	76.9 ± 11.6	84.1 ± 20.9	0.095
Thrombus burden-no. (%)			0.643
0	2 (3.3)	6 (10)	
1	6 (10)	10 (16.7)	
2	4 (6.7)	6 (10)	
3	4 (6.7)	6 (10)	
4	6 (10)	2 (3.3)	
5	38 (63.3)	30 (50)	
Laboratory tests			
Total cholesterol-mmol/L	3.8 ± 2	3.9 ± 1.8	0.893
LDL-mmol/L	3.2 ± 0.94	2.9 ± 0.98	0.335
C-reactive protein-mg/L	6.8 ± 19	4.5 ± 6.5	0.560
Creatinine-mmol/L	80.2 ± 20.1	77.6 ± 20.2	0.627
Troponin (CTn)-ng/L	6319.7 ± 3559	6275.4 ± 3451.8	0.961
Ejection fraction (EF)-%	51.8 ± 8.6	53.7 ± 10.1	0.427
Characteristics of the procedural data			
Direct stenting-no. (%)	30 (50)	18 (37.5)	0.114
Thrombectomy-no. (%)	20 (33.3)	10 (16.7)	0.136
Predilation balloon diameter-mm	2.4 ± 0.21	2.4 ± 0.21	0.945
No. of stents-no. (%)	1.13 ± 0.35	1.17 ± 0.38	0.723
Type of drug-eluting stent-no. (%)			0.554
PROMUS Element	56 (93.3)	58 (96.7)	
GuReater	4 (6.7)	2 (3.3)	
Total stent length-mm	33.1 ± 14.5	31.3 ± 14.6	0.640
Stent diameter-mm	3.17 ± 0.29	3.09 ± 0.41	0.418
Inflation time-seconds	35.87 ± 10.38	10.07 ± 7.19	< 0.0001
Inflation pressure-atm	13.47 ± 1.28	13.40 ± 2.23	0.890
Postdilation-no. (%)	5 (16.7)	9 (30)	0.222
Medication use-no. (%)			
Glycoprotein IIb/IIIa inhibitor	32 (53.3)	30 (50)	0.796
Nitroprusside	0 (0)	4 (6.7)	0.492
Diltiazem	2 (3.3)	4 (6.7)	0.554

*ACEI* angiotensin-converting enzyme inhibitor, *ARB* angiotensin receptor blocker, *CCB* calcium channel block, *TIMI* thrombolysis in myocardial infarction, *LDL* low density lipoprotein, *PBSG* prolonged balloon inflation in stent deployment strategy group, *CDSG* conventional deployment strategy group, *EF* ejection fraction (the EF was measured by modified Simpson's method on transthoracic echocardiography beside the bed when patients in the emergency department before coronary angiography)

### Procedural outcomes

#### Primary outcome

The proportion of patients with an immediate TIMI flow grade < 3 was lower in the PBSG than that in the CDSG after stent deployment (3.3% vs. 36.6%, respectively, *P* = 0.005) (Table 2). The no-reflow proportion of patients was lower in the PBSG than that in the CDSG (0% vs. 30%, respectively, *P* = 0.002). The corrected TIMI frame count was lower in the PBSG than that in the CDSG (35.6 ± 14.5 vs. 49.18 ± 25.2, respectively, *P* = 0.014), and more patients in the PBSG had ST-segment resolution ≥ 50% than that in the CDSG (90% vs. 66.7%, respectively, *P* = 0.028). The percentage of patients who achieved an MBG of 3 was higher in the PBSG than that in the CDSG (60% vs. 20%, *P* = 0.001).

**Table 2:** Primary and secondary outcomes

Outcome	PBSG (n = 60)	CDSG (n = 60)	*P* value
*The primary outcomes*			
The incidence of no-reflow-no. (%)	0 (0)	18 (30)	0.002
Pre-PCI TIMI flow-no. (%)			0.193
TIMI 0–1	50 (83.3)	38 (63.3)	
TIMI 2	4 (6.8)	6 (10)	
TIMI 3	6 (10)	16 (26.7)	
Immediate after PCI TIMI flow-no. (%)			0.005
TIMI 0–1	0 (0)	2 (3.3)	
TIMI 2	2 (3.3)	20 (33.3)	
TIMI 3	58 (96.7)	38 (63.3)	
Myocardial blush grade after PCI-no. (%)			0.001
0	0 (0)	0 (0)	
1	0 (0)	12 (20)	
2	24 (40)	36 (60)	
3	36 (60)	12 (20)	
ST-segment resolution ≥ 50%-no. (%)	54 (90)	40 (66.7)	0.028
Corrected TIMI frame count-no. (%)	35.6 ± 14.5	49.18 ± 25.2	0.014
*The secondary outcomes*			
30-day clinical outcomes-no. (%)	**4 (13.3)**	**7 (23.3)**	**0.506**
Target vessel revascularization	2 (6.7)	3 (10)	1.000
Recurrent myocardial infarction	1(3.3)	3 (10)	0.612
Cardiovascular mortality	1 (3.3)	1 (3.3)	1.00
One-year clinical outcomes-no. (%)	**7 (23.3)**	**11 (36.7)**	**0.399**
Target vessel revascularization	3 (10)	5 (16.7)	0.706
Recurrent myocardial infarction	3 (10)	4 (13.3)	1.00
Cardiovascular mortality	1 (3.3)	2 (6.7)	1.00
Others			
Distal embolization of culprit vessels-no. (%)	0 (0)	2 (3.3)	0.313
Procedure time-min	37 ± 11.8	39 ± 18	0.572
Radiation exposure time-min	1208.6 ± 757.7	932.69 ± 452.9	0.130
Bleeding events-no. (%)	0 (0)	2 (3.3)	0.313

#### Secondary outcome

**Fig. 2:**
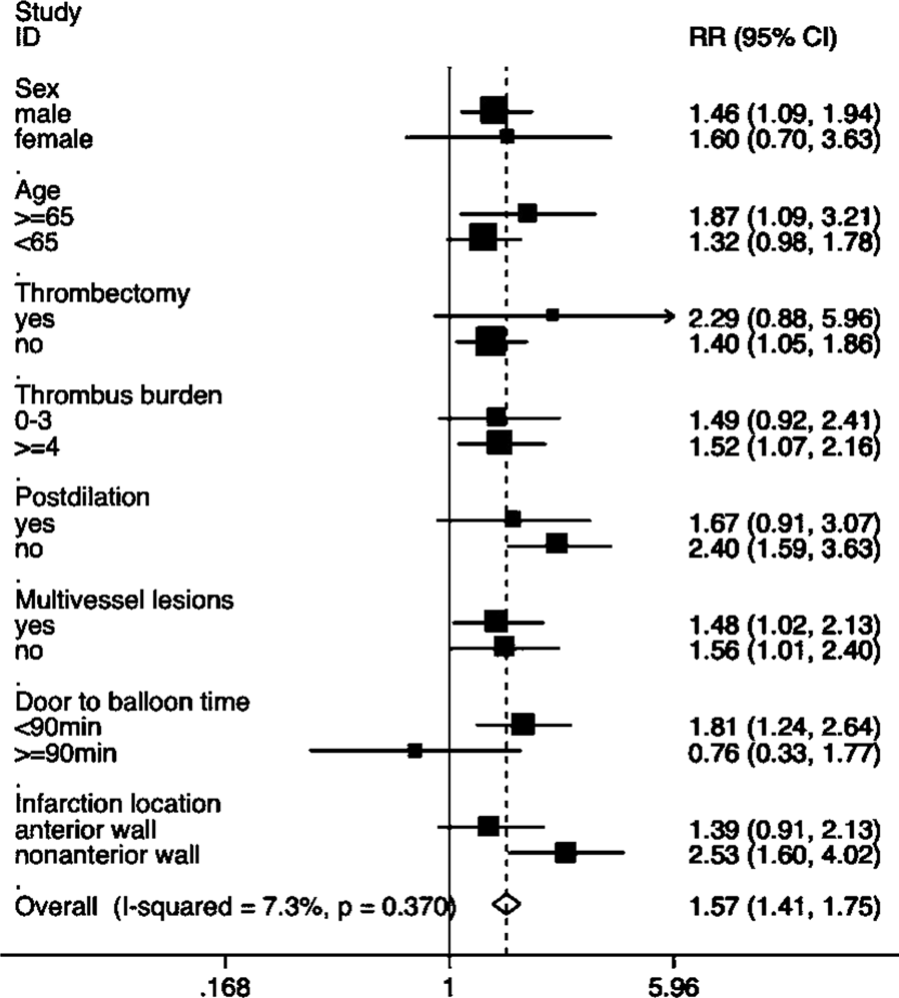
Forest plot of subset analysis

**Fig. 3:**
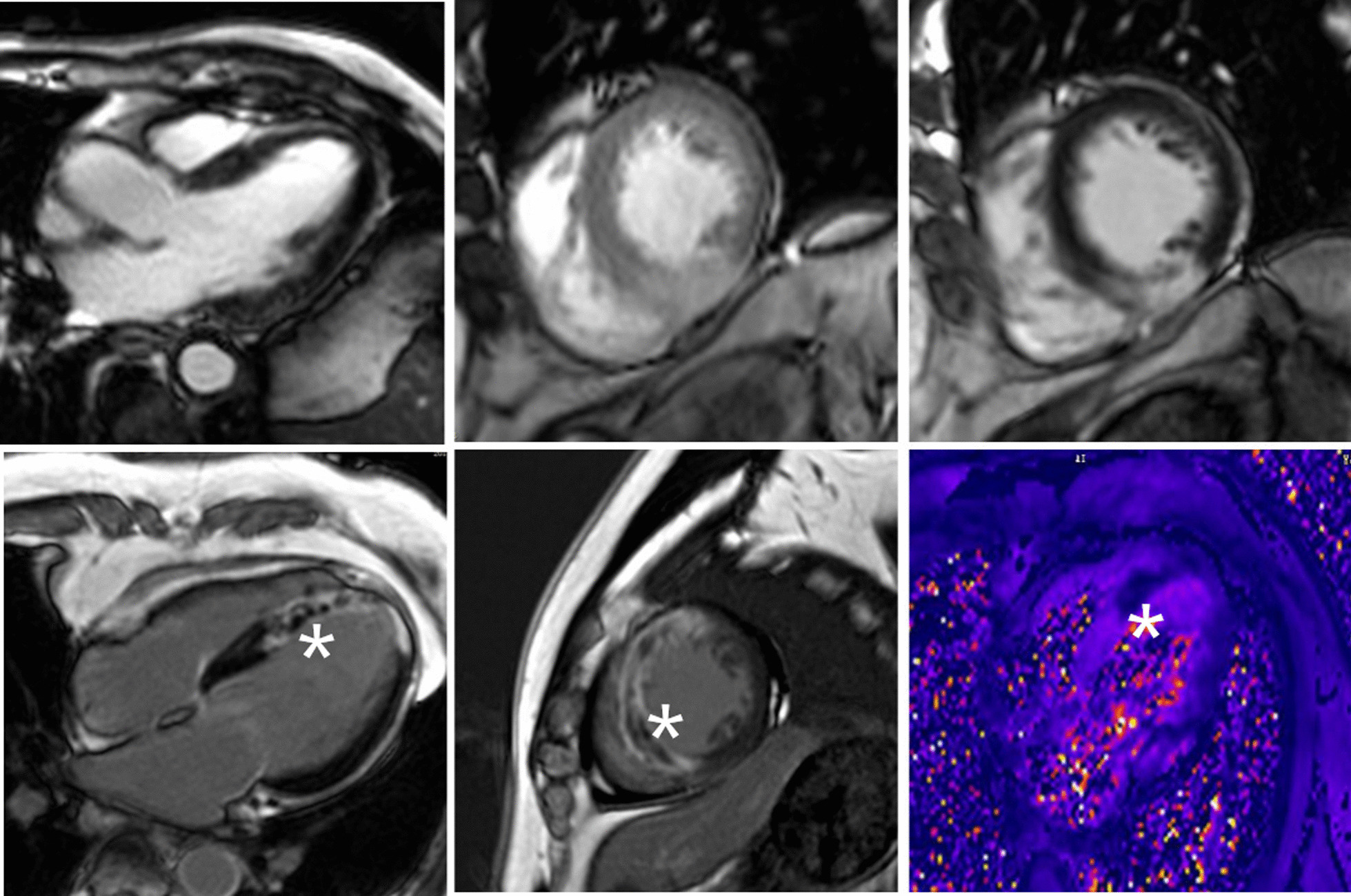
Cardiac magnetic resonance image. The top row was from a patient who under prolonged balloon inflation during stent deployment strategy, there is no imaging features of microvascular obstruction (MVO). Bottom row was from a patient with anteroseptal infarct who under conventional deployment strategy, there is imaging features of MVO (asterisk *)

There was no significant difference in the procedure time, radiation exposure time, or contrast volume between the two groups. The number of bleeding events and clinical end points were also not significantly different between the two groups (Table 2). The result of the subgroup analysis (Fig. 2) showed that male patients (RR = 1.46; 95% confidence interval [CI] = 1.09–1.94), non-thrombectomy (RR = 1.40; 95% CI = 1.05–1.86), high thrombus burden (RR = 1.52; 95% CI = 1.07–2.16), door-to-balloon time < 90 min (RR = 1.81; 95% CI = 1.24–2.64), and non-anterior wall infarction (RR = 2.53; 95% CI = 1.60–4.02) had a lower risk for no-reflow phenomenon, which favors PBSG (P for interaction = 0.07). According to the CMR subset, the PBSG can reduce the incidence of MVO and improve cardiac function (Table 3, Fig. 3).

**Table 3:** Cardiac magnetic resonance (CMR) data

Endpoint	PBSG (n = 30)	CDSG (n = 20)	*P* value
Infarct size (%LVM)	12.1 (4.5)	13.4 (3.9)	0.512
Presence of MVO-no. (%)	2/30 (6.7)	10/20 (50)	0.023
Presence of IMH-no. (%)	6/30 (20)	8/20 (25)	0.378
Myocardial salvage index	60.5 (22.1)	56.4 (20.1)	0.081
LVEF (%)	50.2 (7.5)	42.1 (6.3)	0.026

*%LVM* percentage of LV mass, *MVO* microvascular obstruction, *IMH* intramyocardial hemorrhage, *LVEF* left ventricular ejection fraction (The LVEF was calculated by subtracting the volume at end systole from the volume at end diastole and dividing the result by the volume at end diastole)

## Discussion

To the best of our knowledge, this study is the first to investigate the effect of prolonged balloon inflation during stent deployment strategy in PPCI. We designed this proof-of-concept pilot study to determine whether prolonged balloon inflation during stent deployment or rapid balloon inflation strategies could provide more short- and long-term benefits for patients with STEMI. The major findings of the present study were as follows: (1) the prolonged balloon inflation strategy lessened the occurrence of the no-reflow phenomenon and improved the myocardial microcirculation perfusion, but the difference between 30-day and 1 year clinical outcomes is not observed; (2) there were no significant differences between the study groups in terms of the procedure time, radiation exposure time, and number of bleeding events; (3) according to the subset analysis, it seems that the prolonged balloon inflation strategy was more effective in patients who were male and had a thrombectomy, heavy thrombosis burden, and non-anterior wall infraction; and (4) as evaluated by the CMR subset, the prolonged balloon inflation strategy can reduce the incidence of MVO and improve cardiac function.

In the early days of coronary angioplasty, an auto perfusion balloon angioplasty catheter was used for long balloon inflation, which was based on the hypothesis that the loose flap of dissection may be definitively “tacked back” and might deliver a better result [22–24]. Another study found that approximately half of patients whose initial angioplasty failed could be treated by prolonged balloon dilation [25]. With the wide application of stents, this technique was not used. Subsequently, an in vitro study investigated the duration of balloon inflation for optimal stent deployment [10, 11]. Prolonged balloon inflation could have a significant impact on stent expansion and strut apposition, as evaluated by intravascular ultrasound or optical coherence tomography [26–28]. However, these studies were not limited to patients with STEMI.

Despite the use of PPCI and the development of emergency transports resulting in shorter ischemic times, STEMI-related mortality remains considerable [29]. The no-reflow phenomenon can offset, at least in part, the benefits of PPCI [30]. Coronary microvascular dysfunction plays a key role in the development of the no-reflow phenomenon [31], which occurs in approximately 50% of patients with STEMI after PPCI [21]. Thrombotic material and thromboembolisms will distally travel, either spontaneously or at the time of PPCI, when an angioplasty balloon is expanded or a stent is deployed [3]. The debris released from a PPCI can also include soluble substances, such as vasoactive agents or inflammatory mediators that may stimulate microvascular arteriolar spasms [32, 33]. Thus, removing an intravascular thrombus using an extraction catheter during the procedure to reduce atherothrombotic embolization is an active strategy that has been evaluated in RCT and meta-analysis [34]. Although there were negative results from the TASTE and TOTAL trials and recent guidelines no longer recommend routine aspiration for STEMI [35, 36], neither study have allowed us to rule out the possibility that thrombus aspiration might be beneficial in high-risk patients [37, 38]. However, with more than 30% of patients showing minimal particles after thrombectomy and persistent residual thrombus in in vitro models, distal embolization may not fully prevent thrombectomy [39, 40]. Direct stenting is another strategy to reduce the risk of distal embolization of thrombus fragments [41, 42]. A meta-analysis found that direct stenting significantly reduced the short-term and 1-year mortality and the after-procedural no-reflow phenomenon [43]. However, few of these studies have been limited to patients with STEMI. The Thrombectomy Trialists Collaboration conducted a patient-level meta-analysis and found that direct stenting was not significantly associated with improved clinical outcomes compared with conventional stenting among patients with STEMI undergoing PPCI [44]; approximately 37% of the patients in that study underwent direct stenting [45]. In our study, direct stenting and thrombectomy were performed more often in the PBSG than in the CDSG (50% and 33.3%, respectively), but the differences were not significant. Although the data from the Thrombectomy Trialists Collaboration affirm the facilitation of direct stenting by aspiration thrombectomy, there was no significant interaction between thrombus aspiration and any of the clinical outcome variables investigated [45]. According to our subset analysis, patients with thrombectomy and a heavy thrombosis burden may have better angiographic myocardial reperfusion outcomes, but there was not enough statistical power to confirm this, and the interaction was not excluded.

In the present study, prolonged balloon inflation during stent deployment may work through the following mechanisms. First, a long balloon inflation time with low pressure could reduce the iatrogenic rupture of thin-cap fibroatheroma through stent expansion, which induces distal embolization, particularly in lesions with low-echoic structures and large amounts of atherosclerotic plaque with vulnerable components. Second, although thrombectomy may reduce the thrombus burden, persistent residual thrombus may also cause distal embolization [39]. When added to direct stenting, the loosened atherothrombus material was affixed to the vessel wall under the stent struts. However, incomplete stent deployment and inadequate apposition may weaken the “confinement effect” for atherothrombus material. Therefore, the conventional rapid balloon inflation/deflation strategy was not enough [10], especially in the PPCI setting in which postdilation should be avoided. Third, prolonged balloon inflation may be analogous to the postcondition that was performed within one minute of reflow by brief episodes of reversible ischemia reperfusion during angioplasty. A meta-analysis found that postconditioning following PPCI induced by transient coronary ischemia in patients with STEMI may reduce myocardial injury biomarkers and improve cardiac function [46]. However, the POST and DANAMI 3-iPOST trials found that ischemic postconditioning did not improve myocardial reperfusion and clinical outcomes [47, 48]. The primary end point results contribute to the theory that the postconditioning algorithm, in which postconditioning is instituted after a very short reperfusion time, might be a more effective method. In the present study, if the occlusion persisted for more than 30 s after the stent was deployed, the conditioning therapy was completed before reperfusion injury could occur. Therefore, this postconditioning algorithm may be effective.

The indices of angiography for evaluating coronary microvascular circulation in the cardiac catheterization laboratory are semiquantitative and can be subjective. CMR is the gold standard when assessing the MVO, MI size, LV volume, and LVEF, but it is expensive and not yet widely available. The areas of no-reflow have been associated with MVO, as seen on magnetic resonance images, and correlate with a higher degree of myocardial damage. Therefore, in the present study, we designed a subset of CMR to assess the no-reflow phenomenon after PPCI. Approximately 50% of the patients included in this study underwent CMR. The incidence of MVO was 50% in the CDSG and was consistent with previous studies [49]. The prolonged balloon inflation strategy could reduce the incidence of MVO and improve cardiac function, but not infarction size, as evaluated by CMR. The no-reflow phenomenon is associated with poor healing of the infarct scar, including thinner scars and more infarct expansion; therefore, it appears that some therapies can reduce no-reflow without reducing infarct size [50].

## Limitations

This study has the following limitations. Firstly, the study represents a single-center experience using surrogate end points with a relatively small sample that was underpowered to detect significant differences in the clinical end points. However, this trial implemented rigorous inclusion and exclusion criteria to reduce the risk of confounding bias. The primary outcome (the incidence of the no-reflow phenomenon) was well correlated with subsequent mortality in patients with STEMI. Even when transient, no-reflow during PPCI was associated with poor short- and long-term outcomes. In the future, large randomized clinical trials are necessary to confirm the efficacy of this new approach. Second, because high-risk patients with more complex lesions and unstable hemodynamics were excluded, the results cannot be applied to all patients with STEMI. Third, there may be selection bias in the CMR subset group because there was no randomization between the groups. Because limited by research funding, a randomization was not performed, and some patients did not obtain informed consent. Hence, the results of the CMR subset group should be interpreted with caution. Nonetheless, our study was the first to evaluate this novel approach for STEMI, which can contribute to the value of our trial. Fourth, the dual antiplatelet therapy (DAPT) is vital in patients with acute STEMI undergoing PPCI. But recently, an individual patient level meta-analysis found that aspirin cessation from 1 to 3 months after coronary revascularization and continuation with P2Y12 inhibitor monotherapy may be warranted instead of continuation of DAPT, especially it was associated with lower rates of major bleeding compared with DAPT [51]. In present study, the antiplatelet strategies did not detailed description in baseline characteristics and following up after PCI, this may be a confounding factor for 1 year clinical outcomes. At the same time, whether this treatment strategy has an impact on no reflow phenomenon has not been included in this study, this is one of the limitations in our study.

## Conclusions

In our pilot trial, prolonged balloon inflation during stent deployment strategy during PPCI could lessen the occurrence of the no-reflow phenomenon and improve the myocardial microcirculation perfusion in patients with STEMI. However, these data are preliminary, and large-sample RCTs with a long-term follow-up period are needed to confirm this result.

## Data Availability

The dataset analyzed in the current study is not publicly available because of the lack of consent from the study participants, but it is available from the corresponding author on reasonable request for researchers who meet the criteria for access to confidential data.
